# Moesin Is a Novel Biomarker of Endothelial Injury in Sepsis

**DOI:** 10.1155/2021/6695679

**Published:** 2021-02-13

**Authors:** Yikun Chen, Jiajia Wang, Lei Zhang, Jianjie Zhu, Yuanyuan Zeng, Jian-an Huang

**Affiliations:** ^1^Department of Respiratory Medicine, The First Affiliated Hospital of Soochow University, 215006 Suzhou, Jiangsu Province, China; ^2^Department of Endocrinology and Metabolism, Xinghua People's Hospital Affiliated to Kangda College of Nanjing Medical University, 419 Yingwu Road, Xinghua, 225700 Jiangsu, China

## Abstract

**Objective:**

Increased vascular permeability and inflammation are principal hallmark of sepsis. Moesin (MSN) is a membrane-associated cytoskeleton protein and crucial for the vascular endothelial function. This study is aimed at evaluating the role of MSN in endothelial injury during the process of sepsis.

**Methods:**

Serum MSN in septic patients was measured by ELISA. BALB/c mice were injected with different doses of lipopolysaccharide (LPS) or underwent cecal ligation and single or double puncture (CLP) to mimic sublethal and lethal sepsis. After treatment, their serum MSN and PCT levels, wet to dry lung weights (W/D ratio), bronchoalveolar lavage fluid (BALF) protein concentrations, and lung injury scores were measured. The impact of MSN silencing on LPS-altered Rock1/myosin light chain (MLC), NF-*κ*B, and inflammatory factors in human microvascular endothelial cells (HMECs), as well as monolayer HMEC permeability, was tested in vitro.

**Results:**

Compared with healthy controls, serum MSN increased in septic patients and was positively correlated with SOFA scores and serum PCT levels in septic patients. LPS injection significantly increased serum the MSN and PCT expression, BALF protein levels, and W/D ratio, and the serum MSN levels were positively correlated with serum PCT, lung W/D ratio, and lung injury scores in mice. Similar results were obtained in the way of CLP modelling. LPS enhanced MSN, MLC, NF-*κ*B phosphorylation, increased Rock1 expression, and inflammatory factors release in the cultured HMECs, while MSN silencing significantly mitigated the LPS-induced Rock1 and inflammatory factor expression, NF-*κ*B, and MLC phosphorylation as well as the monolayer hyperpermeability in HMECs.

**Conclusions:**

Increased serum MSN contributes to the sepsis-related endothelium damages by activating the Rock1/MLC and NF-*κ*B signaling and may be a potential biomarker for evaluating the severity of sepsis.

## 1. Introduction

Sepsis is a leading cause of high morbidity and mortality worldwide, and it can lead to dysregulated host responses to infection, even acute multiple organ failure [1]. Currently, treatment for sepsis is very limited, and the high morbidity and mortality rates of sepsis result in high socioeconomic burdens worldwide [2].

Increased vascular permeability is a principal hallmark of sepsis. The endothelial integrity is crucial for the physiological organ function. During the process of infection and sepsis, endothelial cells can initiate and propagate the inflammatory response, and systemic activation of the endothelium will disrupt the endothelium barrier [3, 4]. Such wide spreading vascular dysfunction finally leads to multiple organ failure and death. However, there is no reliable measure biomarker to evaluate the endothelium injury. Therefore, identification of biomarkers for evaluating endothelial activation and injury will be of significance in early management of septic patients.

Moesin, a member of the ezrin-radixin-moesin family, functions to link the plasma membranes to actin-based cytoskeleton and is crucial for the vascular endothelial function. Although three ERM proteins share high amino acid similarity, their expression is organ- and tissue-specific [2]. MSN is predominantly expressed in vascular endothelial cells. Previous studies have found that some factors, such as TNF-*α*, thrombin, and advanced glycation end products, can activate vascular endothelial cells and phosphorylate MSN to increase endothelial cell permeability [5–7]. Furthermore, lipopolysaccharide (LPS) can stimulate endothelial cells to secrete MSN [8]. In addition, MSN is required for the high-mobility group box- (HMGB-) induced endothelial cell hyperpermeability and inflammatory responses, and high levels of blood MSN are detected in septic mice and patients [9]. Hence, MSN participates in the pathogenesis of sepsis. However, little is known on whether MSN can be a useful biomarker for diagnosis and prognosis of sepsis.

In this study, we tested the hypothesis that MSN might be a potential biomarker for evaluating the severity of endothelium damages during the process of sepsis. We examined the MSN expression in septic mice and patients and explored its function in human microvascular endothelial cells (HMECs) in vitro.

## 2. Materials and Methods

### 2.1. Subjects

A total of 46 septic patients were recruited in the Department of ICU in the First Affiliated Hospital of Soochow University and Affiliated Hospital of Jiangsu University from September 2019 to August 2020. Another 24 age- and gender-matched healthy subjects were recruited in the physical examination center of these hospitals. Individual patients with sepsis were diagnosed, based on the Third International Consensus Definitions for Sepsis and Septic Shock [1], and their Sequential Organ Failure Assessment (SOFA) was scored. Unfortunately, three patients were excluded because of over age or malignant tumor. Finally, 20 patients with sepsis and 23 patients with septic shock were included in the study. The exclusion criteria for all participants were the following: <18 or >80 y, cancer, pregnancy, and chronic infection for more than 2 weeks. The study was performed in accordance with the Helsinki Declaration, and the protocol was approved by the Ethics Committee of the First Affiliated Hospital of Soochow University and Affiliated Hospital of Jiangsu University. Individual subjects signed a written informed consent. The demographic and clinical characteristics of these subjects are shown in Table 1. Their peripheral venous blood samples were collected, and their sera were prepared. The levels of serum MSN in individual patients were measured by ELISA.

### 2.2. Animal Experiments

Male BALB/c mice (6-8 weeks old, 20 ± 0.7 g) were purchased from the Animal Research Center of Jiangsu University and kept in a specific pathogen-free facility. The animal experiments were approved by the Animal Care and Use Committee of Soochow University.

The mice were randomized and injected intraperitoneally with vehicle as the control or 3 mg/kg (low-dose group) or 6 mg/kg (high-dose group) LPS daily for two consecutive days (*n* = 12 per group) to induce sepsis [10, 11]. At the end of the experiment, their lungs were harvested. The right lungs of 4 mice were immediately frozen in liquid nitrogen and stored at −80°C, while their left lungs were prepared for histological analysis. Other mice were subjected to bronchoalveolar lavage with 3 ml PBS (1 ml, 3 times) through a tracheal cannula, and the collected BALF samples were centrifuged. The BALF supernatants were stored at -80°C, and the pelleted cells were used for further analysis. The protein concentrations in individual BALF samples were determined by the bicinchoninic acid (BCA) assay using a BCA kit (KeyGen Biotech, Nanjing, China), according to the manufacturer's instruction.

Sublethal sepsis was induced by cecal ligation and puncture (CLP), single puncture. Cecal ligation and double puncture (2CLP) were used to induce lethal sepsis. Mice underwent CLP or 2CLP with an 18-gauge needle as previously described [12, 13]. Sham-operated animals served as controls. There were 20 mice in each group.

### 2.3. Cell Culture and siRNA Transfection

HMECs were purchased from the Fuheng Cell Center (Shanghai, China) and cultured in endothelial cell medium (ScienCell, Los Angeles, USA). HMECs (2 × 10^5^ cells/well) were cultured in six-well plates and when the cells reached about 60% confluency, they were transfected with MSN-specific siRNA or control siRNA (GenePharma, Shanghai, China) using the siRNA transfection Lip3000 kit (Promega) following the manufacturer's instructions. Two days after transfection, the levels of the MSN expression in the transfected cells were analyzed by Western blot and quantitative RT-PCR. The primers sequences of MSN were siNC: sense 5′-UUCUCCGAACGUGUCACGUTT-3′ and three siRNAs: siRNA-1: 5′-GGAUCUUGGCCUUGUGCAUTT-3′, siRNA-2: 5′-GGGCUGAUGCUAUGGCCAATT-3′, and siRNA-3: 5′-GCGACUGGGCCGAGACAAATT-3′.

### 2.4. Histopathology and Immunohistochemical Staining

The collected lung tissues were fixed with 4% paraformaldehyde and paraffin-embedded. The lung tissue sections (4 *μ*m) were routine-stained with hematoxylin-eosin (H&E). The degrees of lung injury were scored in a blinded manner as previously described [14]. To further evaluate the expression of MSN, lung tissues were used to perform immunohistochemistry. Sections were incubated with a primary antibody to MSN (1 : 100 dilution; Abcam, USA) for 1 h at room temperature and then incubated with biotinylated secondary antibodies. Horseradish peroxidase-conjugated IHC Detection Reagent was used to detect antibody-antigen complexes (Cell Signaling Technology). Sections were then developed by the DAB Kit (BD Bioscience, San Jose, CA, USA) and counterstained using Harris hematoxylin.

### 2.5. Measurements of the Lung Wet/Dry Ratio

The dissected right lungs were immediately weighed and dried at 65°C in an oven for 48 h. The dried lung tissues were weighed. The ratios of dry-to-wet lung weights were calculated.

### 2.6. Enzyme-Linked Immunosorbent Assay (ELISA)

The levels of human (SEC642Hu) and mouse serum MSN (SEC642Mu) and PCT (SEA689Mu) were quantified by ELISA using specific kits (USCN Life Sciences, Wuhan, China), according to the manufacturer's instructions. To explore the secretion mechanisms of MSN in HMECs after LPS stimulation, three inhibitors of the main pathways of protein secretion were employed in the experiment. The dosage of inhibitors is as follows: the autophagy inhibitor 3-methyladenine (3-MA, 1 mM, Selleck, S2767), the exosome secretion inhibitor 5-(N,N-dimethyl)-amiloride DMA (DMA, 50 nM, APExBIO, C3505), and the protein transport inhibitor brefeldin A (BFA, 10 ng/ml, APExBIO, B1400). The supernatants were collected from the cultured medium 24 h later. To explore the role of MSN in the process of excessive inflammation caused by sepsis, HMECs were, respectively, transfected with siNC and siMSN RNA for 48 h, then exposed to LPS for 24 h, and the supernatants were collected to measure IL-1*β*, IL-18, IL-6, and TNF-*α* levels (E-EL-H0149c, IL-18 E-EL-H0253c, E-EL-H0102c, E-EL-H0109c, Elabscience Biotechnology, Wuhan, China).

### 2.7. Western Blotting

The collected lung tissues were homogenized, and HMSCs were lyzed. After quantified the protein concentrations, the lung tissue homogenates, and cell lysates (30 *μ*g/lane) were separated by 10% sodium dodecyl sulfate polyacrylamide gel eletrophoresis (SDS-PAGE) on 12% gels and transferred onto polyvinylidene difluoride (PVDF) membranes. After being blocked in 5% skim milk in TBST, the membranes were probed with the primary antibodies overnight at 4°C. The bound antibodies were detected with horseradish peroxidase–labeled secondary antibodies and visualized with enhanced chemiluminescent reagents (Millipore). The primary antibodies included anti-MSN and anti-phospho T558-MSN (Abcam), anti-Rock1 (Abcam), anti-p65 and anti-phospho Ser536-p65 (Cell Signaling Technology), myosin light chain 2, and phosphomyosin light chain 2 (pMLC2, Thr18/Ser19, Cell Signaling Technology) (1 : 1000 for all).

### 2.8. Coimmunoprecipitation

HMECs were lysed with modified RIPA buffer containing protease and phosphatase inhibitor cocktail for 30 min. Cell lysates were collected and centrifuged at 10,000 g for 10 min at 4°C. The supernatants were collected, and immune complexes were captured by protein G beads slurry, according to the manufacturer's instructions. Immunoprecipitation was performed with antibodies to Rock1. The beads were eluted with RIPA buffer and boiled in 2× SDS protein loading buffer for 7 min. Then, samples were analyzed by Western blot.

### 2.9. Quantitative Real-Time PCR

Total RNA was extracted from HMECs using the RNAiso Plus kit (Takara, Kusatsu, Shiga, Japan), and RNA samples (1 *μ*g each) were reversely transcribed into cDNA using the M-MLV First Strand kit (Life Technologies, Gaithersburg, MD, USA). The relative levels of MSN to control *β*-actin mRNA transcripts were quantified by qRT-PCR using ×2 SYBR-Green qPCR SuperMix (High ROX) (Bimake, Houston, USA) and specific primers (Sanon Biotech, Shanghai, China) in the ABI StepOne Plus RealTime PCR system (Applied Biosystems). The PCR reactions were performed in triplicate. The sequences of primers for MSN were forward 5′-GATGCTGTCCTGGAATATCTGA-3′ and reverse 5′-TCTGCTCATAGATGTTGAGACC-3′. Sequences of primers for GAPDH were forward 5′-UGACCUCAACUACAUGGUUTT-3′ and reverse 5′-AACCAUGUAGUUGAGGUCATT-3′. The data were analyzed by the 2^-*ΔΔ*Ct^ method.

### 2.10. Measurement of Endothelial Permeability

The impact of the MSN expression on the permeability of monolayer HMECs was determined by a dextran penetration assay. Briefly, the different groups of HMECs (5 × 10^4^ cells/well) were cultured in the inserted chambers (4 *μ*m pore size and 12 mm diameter) in 12-well transwell plates for 2 days, and when the cells reached confluence, the cells in individual wells were treated in triplicate with LPS (0, 1, 2, and 4 *μ*g/ml) for 24 h. After being washed with PBS, the cells in the inserted chambers were cultured in the bottom chamber with fresh medium and treated with 0.5 mg/ml fluorescein isothiocyanate- (FITC-) labeled dextran (40 Kd, Sigma-Aldich) at 37°C for 30 minutes. The fluorescent signals in the supernatants of the bottom chambers were determined by spectrophotometry at (Ex (*λ*) 490 nm; Em (*λ*) 520 nm). The data in the experimental groups were normalized to that of the control group.

### 2.11. Statistical Analysis

Data are expressed as mean ± SD or case numbers. The difference between groups was analyzed by the independent *t*-test or Mann–Whitney *U* test. The difference among groups was analyzed by one-way ANOVA, posthoc Kruskal-Wallis test (non-normal distributed parameters), and Bonferroni correction (normally distributed parameters). The relationship between two variables was analyzed by linear or logistic regression correlation analysis. All statistical analyses were performed using the SPSS version 23 and GraphPad Prism 7 Software. A *p* value of <0.05 was considered statistically significant.

## 3. Results

### 3.1. Increased Serum MSN Levels Are Positively Correlated with the Severity of Sepsis in Patients

**Figure 1:**
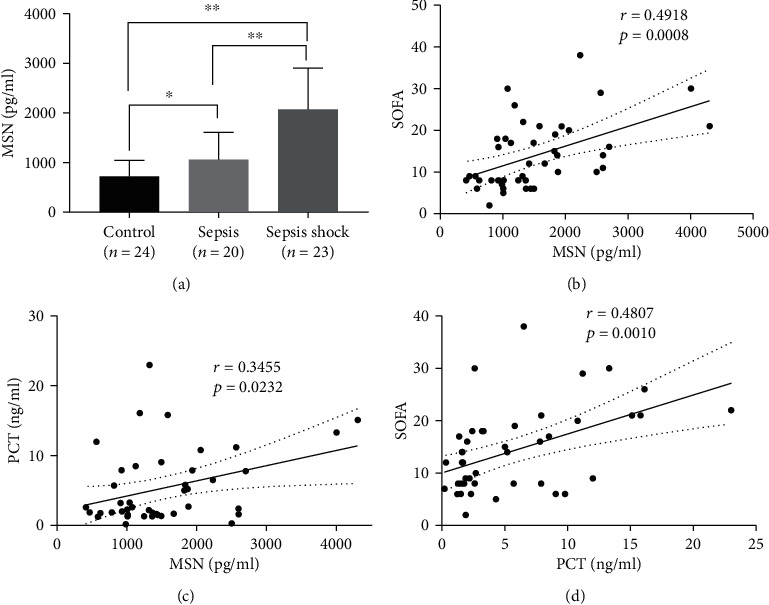
Increased serum MSN levels are positively correlated with SOFA scores and PCT levels in septic patients. (a) Serum MSN levels in septic patients and healthy controls were measured by ELISA. (b, c) The correlation between serum MSN levels and SOFA scores or PCT levels in septic patients. (d) The correlation between serum PCT levels and SOFA scores in septic patients. ^∗^*p* < 0.05, ^∗∗^*p* < 0.01.

To understand the potential value of serum MSN, we recruited 20 septic patients, 23 septic shock patients, and 24 age- and gender-matched healthy subjects, and we measured their serum MSN by ELISA. We found that the serum MSN levels in septic shock patients (1974.57 ± 838.15 pg/ml) were significantly higher than that in the sepsis group (1038.55 ± 477.19 pg/ml) and the healthy controls (726.67 ± 64.28 pg/ml, *p* < 0.01 for both, Figure 1(a)). Furthermore, the levels of serum MSN in the septic patients were also significantly higher than that in the healthy subjects (*p* = 0.0278) (Figure 1(a)). Correlation analysis revealed that the levels of serum MSN in the septic patients were positively correlated with SOFA scores (*r* = 0.4918, *p* = 0.0008, Figure 1(b)) and serum PCT levels (*r* = 0.3455, *p* = 0.0232, Figure 1(c)). As expected, the levels of serum PCT in all patients were positively correlated with SOFA scores (*r* = 0.4807, *p* = 0.0010, Figure 1(d)). The results indicated that increased MSN levels were positively correlated with the severity of sepsis in this population.

### 3.2. Increased Serum MSN Levels Are Correlated with Elevated Lung Permeability in a Mouse Model of LPS-Induced Septic Lung Injury

**Figure 2:**
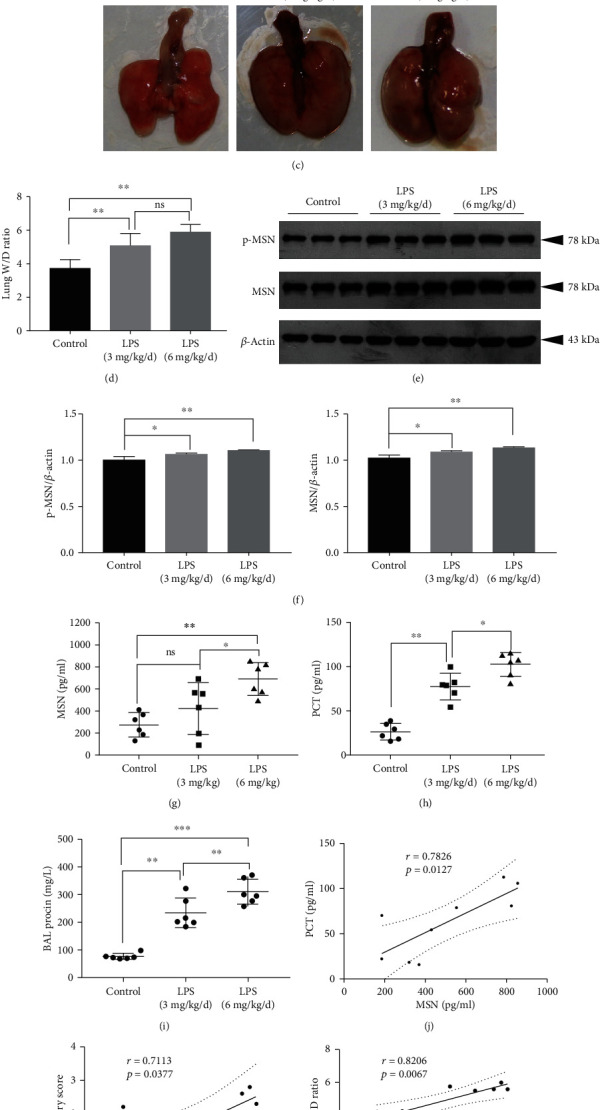
LPS increased the MSN expression and lung injury in mice. BALB/c mice were intraperitoneally injected with the indicated doses of LPS for two days, and their serum MSN and PCT levels were examined by ELISA. Their lung W/D ratios and BALF protein levels were measured. (a) H&E staining of lung tissue sections in different groups of mice (magnification ×100). (b) Lung injury scores. (c) Pulmonary edema in different groups of mice. (d) The lung W/D ratios in different groups of mice. (e) LPS increased p-MSN and MSN expression in lung tissues in different groups of mice, and the semiquantification for WB was shown in (f). Serum MSN (g) and PCT (h) levels in different groups of mice. (i) BALF protein concentrations in different groups of mice. The correlation between serum MSN and PCT (j), serum MSN and lung injury scores (k), serum MSN levels, and lung W/D ratios (l). NS: not significant; ^∗^*p* < 0.05, ^∗∗^*p* < 0.01, ^∗∗∗^*p* < 0.001.

To test whether increased MSN levels could contribute to endothelium damages, a mouse model of septic lung injury was established by injecting different doses of LPS. H&E staining revealed that LPS injection significantly impaired the alveolar microstructure, enhanced inflammatory cell infiltration and lung damages, and increased lung injury scores, particularly in the mice with a higher dose LPS group (Figures 2(a) and 2(b)). There were also obvious edema, congestion, and high W/D ratios in the harvested lung tissues of the septic mice (Figures 2(c) and 2(d)). Then, we further tested the expression of phosphorylated MSN and total MSN in lung tissues by Western blotting, the results show that LPS injection can significantly increase the MSN expression in lung tissues (Figure 2(e)), and the semiquantification for WB was shown in(Figure 2(f)). We also found that LPS injection elevated the serum MSN and PCT levels, particularly in the higher dose LPS group (Figures 2(g) and 2(h)). As expected, injection with LPS also significantly increased the levels of BALF proteins, related to that in the control mice (*p* < 0.01,for both Figure 2(i)). To explore the relationship between MSN and sepsis correlation analysis between MSN and lung injury scores, lung W/D ratios and PCT were performed. Interestingly, the levels of serum MSN were positively correlated with PCT (*r* = 0.7826, *p* = 0.0127, Figure 2(j)), the lung injury scores (*r* = 0.7113, *p* = 0.0377, Figure 2(k)), and the lung W/D ratios (*r* = 0.8206, *p* = 0.0067, Figure 2(l)) in septic animals. Such findings indicated that increased serum MSN levels were indicatives of elevated lung permeability and vascular endothelium damage in septic mice.

### 3.3. Increased Serum MSN Levels Are Correlated with Lung Injury Score and W/D Ratio in a CLP-Induced Septic Mouse Model

**Figure 3:**
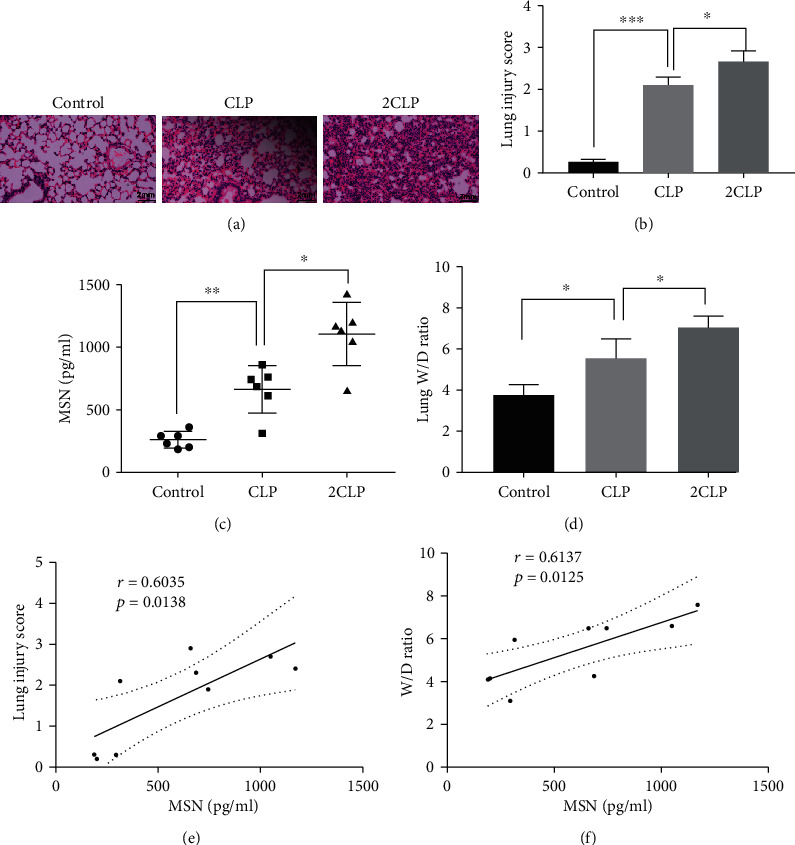
CLP surgery increased the MSN expression and lung injury in mice. Sublethal and lethal CLP were used to construct the septic mouse model, and their serum MSN levels were examined by ELISA, 24 h after CLP surgery. (a) H&E staining of lung tissue sections in different groups of mice (magnification ×100). (b) Lung injury scores. (c) Serum MSN levels in different groups of mice. (d) The W/D ratios in different groups of mice. The correlation between serum MSN and lung injury scores (e), serum MSN levels, and lung W/D ratios (f). NS: not significant, ^∗^*p* < 0.05, ^∗∗^*p* < 0.01, ^∗∗∗^*p* < 0.001.

To further confirm the relationship between MSN and sepsis, we used sublethal and lethal CLP to construct the septic mouse model again. As expected, CLP surgery significantly aggravated the lung pathological damage, particularly in the lethal CLP group (Figure 3(a)). Correspondingly, the lethal CLP group has a high lung injury score than the sublethal CLP group (Figure 3(b), *p* < 0.05). Similarly, we found that the serum MSN increased significantly after CLP surgery, while this increased trend was more obvious in the lethal CLP group (Figure 3(c)). CLP surgery also significantly increased the W/D ratios related to that in the control mice (Figure 3(d), *p* < 0.05). Then, we performed the correlation analysis between MSN and lung injury score and lung W/D ratios. We found that the lung injury scores in CLP mice were positively correlated with serum MSN levels (*r* = 0.6035, *p* = 0.0377, Figure 3(e)) and also have a positive correlation with lung W/D ratios (*r* = 0.6137, *p* = 0.0125, Figure 3(f)).

### 3.4. LPS Upregulates the MSN Expression in HMECs in a Time- and Dose-Dependent Manner

**Figure 4:**
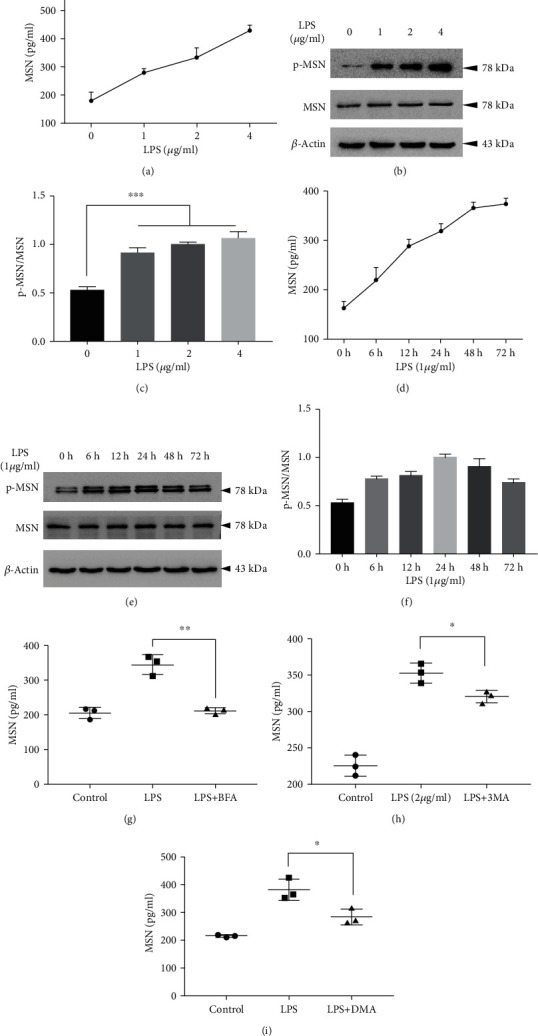
LPS upregulates the MSN expression and phosphorylation in HMECs in a dose- and time-dependent manner. HMECs were treated with different doses (0, 1, 2, and 4 *μ*g/ml) of LPS for 24 h or exposed to LPS (1 *μ*g/ml) up to 72 h. The levels of MSN in the supernatants of cultured cells were measured by ELISA. To explore the secretion mechanisms of moesin in HMECs after LPS stimulation, three inhibitors of the main pathways of protein secretion were employed in the experiment. The dosage of inhibitors is as follows: the autophagy inhibitor 3-methyladenine (3-MA, 1 mM, Selleck, S2767), the exosome secretion inhibitor 5-(N,N-dimethyl)-amiloride DMA (DMA, 50 nM, APExBIO, C3505), and the protein transport inhibitor brefeldin A (BFA, 10 ng/ml, APExBIO, B1400). LPS increased MSN levels in the supernatants of cultured cells in a dose- and time-dependent manner (a, d). The relative levels of the MSN protein expression in cell lysates were determined by Western blotting. (b, e) And the semiquantification for WB was shown in (c, f). MSN secretion was inhibited by 3-MA (h), DMA (i), and BFA (g) to varying degrees. NS: not significant; ^∗^*p* < 0.05, ^∗∗^*p* < 0.01, and ^∗∗∗^*p* < 0.001.

To understand the molecular mechanisms underlying the potential effect of LPS on human endothelial cells, we further tested the effect of LPS on the MSN expression and relevant signaling in vitro. We found that treatment with LPS increased the levels of MSN in the supernatants of cultured HMECs in a dose- and time-dependent manner (Figures 4(a) and 4(d)). Western blot analysis indicated that treatment with LPS did not significantly change the relative levels of the total MSN expression, but significantly enhanced MSN phosphorylation in HMECs, particularly with a higher dose of LPS at 24 h posttreatment (Figures 4(b) and 4(e)), and the semiquantification for WB was shown in (Figures 4(c) and 4(f)). Collectively, LPS treatment increased MSN phosphorylation in HMECs in vitro.

We next explore the secretion manner of MSN. After treatment with 3-MA, DMA, and BFA, respectively, MSN secretion was slightly inhibited by 3-MA and DMA, but significantly inhibited by BFA (Figures 4(h), 4(i), and 4(g)), which suggesting that MSN can be mainly secreted via the Golgi apparatus transport pathway.

### 3.5. MSN Silencing Mitigates the LPS-Increased HMEC Monolayer Permeability In Vitro

**Figure 5:**
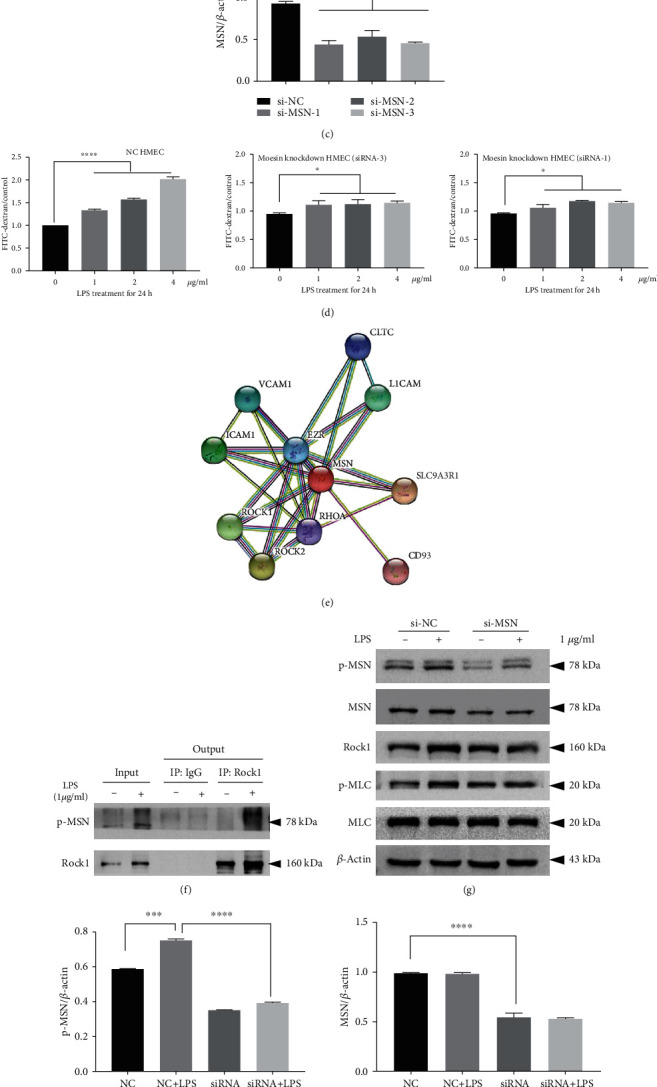
MSN inhibition reversed LPS-induced vascular hyperpermeability and recovered the MLC expression in HMECs via regulating the Rock1 activity.(a) RT-qPCR and (b) Western Blotting analyses of the efficacy of MSN silencing in HMECs, and the semiquantification for MSN silencing was shown in (c). (d) MSN silencing abrogated the LPS-induced vascular hyperpermeability in monolayer HMECs, determined by the FITC-dextran-based barrier integrity assay. (e) Protein interaction analysis showed that MSN was closely related to the RhoA/Rock pathway. (f) Coimmunoprecipitation analyses of the interaction between p-MSN and Rock1 in HMECs treated with or without LPS (24 h). (g) MSN silencing mitigated the LPS-increased MSN and MLC phosphorylation and Rock1 expression in HMECs, and the semiquantification for WB was shown in (h). ^∗^*p* < 0.05, ^∗∗^*p* < 0.01, and ^∗∗∗^*p* < 0.001.

To determine the role of MSN in the LPS-mediated barrier disruption, we generated MSN silencing HMECs by transfection with MSN-specific siRNA. We found that transfection with MSN-specific siRNA significantly reduced the levels of MSN mRNA transcripts and protein expression, demonstrating the efficacy of MSN silencing in HMECs (Figures 5(a) and 5(b)), and the semiquantification for WB was shown in (Figure 5(c)). Among the three siRNAs, siRNA-3 and siRNA-1 showed better knock down effect and were used in the follow-up experiments. While treatment with different doses of LPS significantly increased the amounts of penetrated FITC-dextran in the control siRNA-transfected HMECs in a dose-dependent manner (*p* < 0.001 for all), treatment with the same doses of LPS only slightly increased the levels of penetrated FITC-dextran in the MSN silencing HMECs (*p* < 0.05 for all, Figure 5(d)). Protein interaction analysis and coimmunoprecipitation were used to explore the possible pathway involved in MSN-mediated endothelial barrier breakdown. Protein interaction analysis showed that MSN was closely related to the rhoA/Rock pathway (Figure 5(e)). The coimmunoprecipitation assay was performed, and endogenous interaction of p-MSN-ROCK1 complex was confirmed in HMECs (Figure 5(f)). Western blot analyses revealed that in comparison with that in the control HMECs, MSN silencing not only decreased MSN expression but also significantly mitigated or abrogated the LPS-enhanced MLC phosphorylation and Rock1 expression in HMECs (Figure 5(g)), and the semiquantification for WB was shown in (Figure 5(h)). Such findings indicate that LPS damages the integrity of endothelium, dependent on MSN phosphorylation.

### 3.6. MSN Silencing Attenuates LPS-Induced Inflammatory Cytokine Production

**Figure 6:**
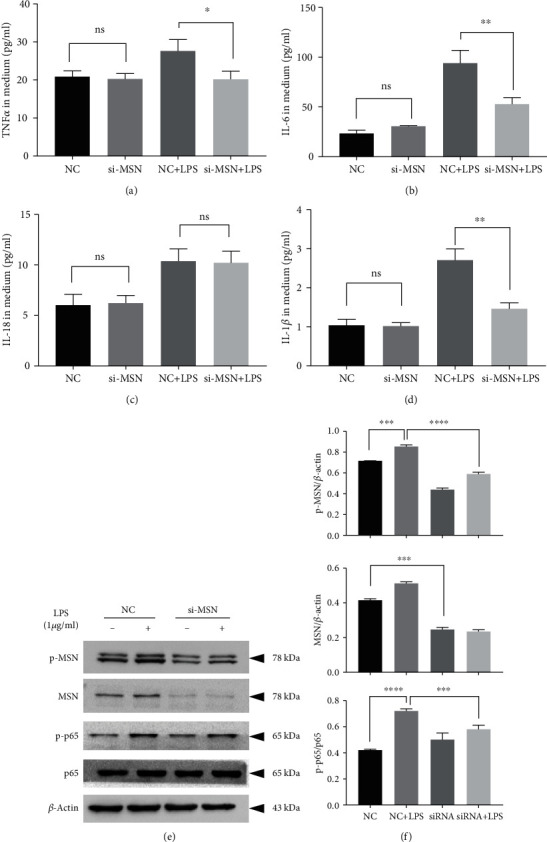
MSN silencing reversed LPS-induced inflammatory cytokine production and p65 phosphorylation in HMECs. HMECs were, respectively, transfected with siNC and siMSN RNA for 48 h, then exposed to LPS for 24 h, and the supernatants were collected for the ELISA test. MSN silencing reduced the production of TNF-*α* (a, *p* < 0.05), IL-6 (b, *p* < 0.01), IL-18 (c, ns), and IL-1*β* (d, *p* < 0.01) in the medium supernatant of HMECs. In addition, MSN silencing also decreased the expression of phosphorylated p65 increased by LPS (e), and the semiquantification for WB was shown in (f).

To further explore the proinflammatory effects of MSN in sepsis, we next performed ELISA to measure the effects of MSN silencing on the production of the inflammatory cytokines TNF-*α*, IL-6, IL-1*β*, and IL-18 in medium supernatant of HMECs. We also detected the expression of the inflammation-related signaling pathway NF-*κ*B by Western blotting. MSN silencing reduced the levels of TNF-*α*, IL-6, IL-18, and IL-1*β* in the medium supernatant of HMECs (*p* < 0.05, *p* < 0.01, ns, *p* < 0.01, respectively) (Figures 6(a)–6(d)). In addition, MSN silencing also decreased the expression of phosphorylated p65 increased by LPS (Figure 6(e)), and the semiquantification for WB was shown in (Figure 6(f)).

## 4. Discussion

Sepsis is a life-threatening organ dysfunction caused by the dysregulated host response to infection, sepsis-3 definition of sepsis with the clinical criteria based on the SOFA score. Septic shock is a special form of sepsis, which refers to a state of acute circulatory failure [15]. Rapid diagnosis is crucial for effective treatment of septic patients. However, there is still no ideal biomarker for diagnosis of sepsis. Although microbiological examination remains as the gold standard for the infection diagnosis, it requires 24 to 72 hours or longer to complete [16]. Serum PCT and C-reactive protein (CRP) levels are widely used as specific biomarkers for diagnosis of bacterial infection-related inflammation, but they are limited in their ability to distinguish sepsis from other inflammatory conditions [17]. The sepsis 3.0 criteria confirm the advantage of the SOFA score in diagnosis and prediction of sepsis. In this study, we observed that increased serum MSN and PCT levels in septic patients were positively correlated with SOFA scores, and both of them have a similar correlation coefficient to SOFA scores. Furthermore, increased serum MSN levels were also correlated positively with W/D ratios and lung pathological damage scores in septic mice. Such findings suggest that serum MSN levels may be a potential biomarker for evaluating the severity of sepsis.

MSN is a protein linker between cellular membranes and the actin-based cytoskeleton in cells. MSN is always maintained in a resting stage, in which its N-terminal and C-terminal domains connect each other to form a closed conformation, leading to its functional sites hidden inside the molecule. MSN phosphorylation changes its conformation into an active conformation by separating its N-terminal and C-terminal domains to expose its functional sites that attach to the cell membrane and cytoskeleton, respectively. The phosphorylated MSN is closely associated with cytoskeletal rearrangement [18–20]. MSN is highly expressed in endothelial cells, including pulmonary vascular and lymphocytes [21]. Both of high vascular endothelium permeability and aberrant macrophage activation are critical events in the pathogenesis of sepsis [22, 23]. We found that LPS increased MSN levels in the supernatants of cultured HMECs, MSN phosphorylation, and the monolayer HMEC permeability. Conversely, MSN silencing by transfection with a specific siRNA mitigated or abrogated the LPS-increased monolayer HMEC permeability in vitro. Our data support the notion that phosphorylated MSN is crucial for high endothelium permeability. Given that MSN can interact with a variety of effector molecules through different mechanisms, increasing endothelium permeability [24], MSN may contribute to the pathogenesis of sepsis and be a potential target for design of therapies for preserving the endothelium integrity. Therefore, our findings may provide new insights into the pathogenesis of sepsis.

It is well known that MSN is important for the innate immune response. A previous study has shown that MSN may act as a receptor of LPS on monocytes [25]. LPS can bind to the carboxyl-terminus of MSN to promote the MSN expression and phosphorylation. The activated MSN is associated with TLR4 and CD14, leading to the LPS-related signaling and proinflammatory cytokine production [26]. Hence, MSN is crucial for the maintenance of vascular endothelial barrier and innate immune cell activation. Consistently, we found that (1) LPS increased MSN levels in the supernatants of cultured HMECs in a dose- and time-dependent manner. Increased serum MSN levels were detected in septic patients, particularly in septic shock patients and septic mice. (2) The levels of serum MSN were positively correlated with BALF protein concentrations and wet-to-dry lung weight ratios, the hallmarks of alveolar capillary barrier damages. (3) The levels of serum MSN were positively correlated with serum PCT levels and pathological lung injury scores in septic mice. (4) MSN knockdown by siRNA significantly inhibited the expression of inflammatory cytokines (TNF-*α*, IL-1*β*, IL-6) in the culture supernatant of HMECs, which may be achieved by inhibiting the NF-*κ*B pathway. These data suggest that activated MSN may be important for the endothelial barrier damages, deteriorating inflammatory responses during the process of sepsis.

Previous studies have shown that activated MSN could translocate to the extracellular surface [27, 28]. In our study, the protein transport inhibitor brefeldin A at the concentration of 10 ng/ml could significantly inhibit MSN secretion, which may partly explain the secretory mechanism of MSN after LPS stimulation.

In our study, we found that LPS significantly enhanced MSN and MLC phosphorylation and Rock1 expression in HMECs, which was in line with increased monolayer HMEC permeability. Furthermore, MSN silencing significantly mitigated or abrogated the LPS-increased MSN and MLC phosphorylation as well as Rock1 expression in HMECs, consistent with the observation in thrombin-induced vascular injury [29]. These findings suggest that activated MSN may upregulate the Rock1 expression, enhancing MLC phosphorylation during the process of septic vascular injury. It is well established that MLC phosphorylation is one of the initial events during gap formation and barrier disruption [30]. The mechanisms by which activated MSN-enhanced MLC phosphorylation remain unclear. Previous studies have shown that MSN appear to act as both upstream [31] and downstream [32] of the Rho family of GTPases, which regulate remodeling of the actin cytoskeleton. Furthermore, Rho can activate Rock1 that can phosphorylate MLC [33]. Actually, activation of Rock1 by thrombin or histamine increases vascular permeability and intercellular gap formation [34]. Consistently, we found that LPS treatment significantly increased the Rock1 expression, paralleling with increased MSN and MLC phosphorylation in HMECs, which were significantly mitigated by MSN silencing. Therefore, MSN is critical for the endothelial barrier disruption by activating the Rock1/MLC signaling in endothelial cells.

In conclusion, our data indicate that increased serum MSN levels are correlated with the SOFA scores and serum PCT levels in septic patients and may be a valuable biomarker for evaluating the severity of sepsis. We recognized that our study had some limitations, such as small sample size, an observational study in one center, and the lack of molecular mechanisms by which activated MSN upregulating the Rock1 expression. Therefore, further perspective studies in multiple centers with a bigger population are necessary to confirm the findings and to investigate the molecular mechanisms underlying the action of MSN during the pathogenic process of sepsis.

## Figures and Tables

**Table 1:** The demographic and clinical characteristics of subjects.

	Control	Sepsis	Sepsis shock	*p* value
Number	24	20	23	—
Gender M/F	16/8	15/5	15/8	0.762
Age (yr)	64.25 ± 10.18	65.85 ± 14.83	71.17 ± 10.49	0.123
MSN (pg/ml)	726.67 ± 64.28	1038.55 ± 477.19	1974.57 ± 838.15	<0.01
PCT (ng/ml)	—	1.85 (0.73, 3.05)	5.20 (1.68, 15.2)	<0.01

## Data Availability

All data used in the paper can be obtain from the correspondence author If there is a proper reason.
